# Q&A: What is biophysics?

**DOI:** 10.1186/1741-7007-9-13

**Published:** 2011-03-02

**Authors:** Huan-Xiang Zhou

**Affiliations:** 1 Department of Physics and Institute of Molecular Biophysics, Florida State University, Tallahassee, FL 32306, USA

## 'Biophysics' implies physics applied to biology: is that what biophysics is?

Yes, biophysics is the study of biological systems and biological processes using physics-based methods or based on physical principles.

## What does physics have to offer biology?

Physics provides the fundamental theories for understanding biomolecules. For example, statistical mechanics, a cornerstone of modern physics, is also the foundation for understanding the behaviors of biomolecular systems. Electron transfer within protein matrices, which drives respiration and photosynthesis, can only be understood with the help of quantum mechanics. In essence, an electron can hop from one position to another within a protein matrix only when the energy levels before and after the hop are equal.

Importantly, many of the powerful tools for investigating biomolecules were initiated by physicists. X-ray crystallography provides a telling example. X-rays were discovered by Wilhelm Röntgen (1901 Nobel Prize in Physics) and their diffraction by crystals was first demonstrated by Max von Laue (1914 Nobel Prize in Physics). The subsequent mathematical formulation of the diffraction pattern by the Braggs, father and son (1915 Nobel Prize in Physics), ushered in the new field of X-ray crystallography. This made possible the determination of the first protein structures by Max Perutz and John Kendrew (1962 Nobel Prize in Chemistry), the structure of DNA by Francis Crick, James Watson, and Maurice Wilkins (1962 Nobel Prize in Physiology or Medicine), and the structures of the photosynthetic reaction center (1988 Nobel Prize in Chemistry), ion channels (2003 Nobel Prize in Chemistry), RNA polymerase II (2006 Nobel Prize in Chemistry), and the ribosome (2009 Nobel Prize in Chemistry). Similar paths can be traced for nuclear magnetic resonance spectroscopy (1943, 1944, and 1962 Nobel Prizes in Physics; 1991 and 2002 Nobel Prizes in Chemistry; and 2003 Nobel Prize in Physiology or Medicine), atomic force microscopy (1986 Nobel Prize in Physics), electron microscopy (1986 Nobel Prize in Physics), and single-molecule techniques such as optical tweezers (1997 Nobel Prize in Physics).

Many computational techniques - for example, molecular dynamics simulation - that are now widely used for modeling biomolecular systems also have their origins in physics.

## I'm a physicist interested in working on biological problems. How do I make the transition?

You are in good company: some of the giants in modern biology, including Max Delbrück, Francis Crick, and Seymour Benzer, made the transition from physics. As Crick learned, you have to adjust from the "'elegance and deep simplicity"' of physics to the "'elaborate chemical mechanisms that natural selection has evolved over billions of years."' As Crick put it, the adjustment is "'almost as if one had to be born again."' You have to take the time to learn the biology. The transition can be eased by collaborating with another biophysicist or a biologist.

However, despite the significant barrier to the transition from physics to biology, intellectually it is probably still far easier than the transition in the opposite direction!

## What are the major contributions of biophysics to modern biology?

An important contribution of biophysicists to modern biology is the perspective that biological processes can be understood from the interactions between and within the constituent molecules. Therefore, the behaviors of biological systems can be predicted from physical principles.

A biological problem that has been mostly tackled by biophysicists is protein folding, by which a nascent polypeptide chain coming off the ribosome finds its unique structure in its native environment. The broad outlines of how the protein avoids the vast number of alternative conformations and quickly finds its native structure are now clear. Some may go as far as claiming the problem is solved. Biophysicists are now using very similar approaches to study the binding of proteins and other biomolecules as well as more complex biological processes.

Biophysicists are largely responsible for dramatic increases in the spatial resolution of structural characterization and the temporal resolution of dynamical characterization, and for bringing the study of biological processes to the single-molecule level.

Biophysicists have demonstrated that many essential features of complex biological systems can be emulated by relatively simple computational models. In particular, artificial neural networks are shown to produce associative memory, an essential function of the brain.

## Some would quibble that that's computational neuroscience, not biophysics, and isn't there some argument about how much such models tell us about the real biology?

How the modeling work is labeled is less important than the fact that it is able to demonstrate that many essential biological features seem generic and robust. That is, they emerge from relatively simple models and are insensitive to details of the models. There are now similar efforts dealing with signaling and gene regulatory networks. Still, a fundamental understanding of these processes will require considering the physical interactions between the molecules involved.

## Have biological problems inspired new physics?

Yes. One example is the theory of complex systems, in which a key concept is emergent properties. These are properties that are not intrinsic to the individual components of a system but are only produced when the components work together as a whole system. For instance, a neural network can produce memory only through the interactions of all the neurons in the network.

In addition, biological problems have stimulated renewed interest in areas like stochastic processes and open, driven systems.

## Doesn't biophysics also embrace physical chemistry and cell biology?

**Figure 1:**
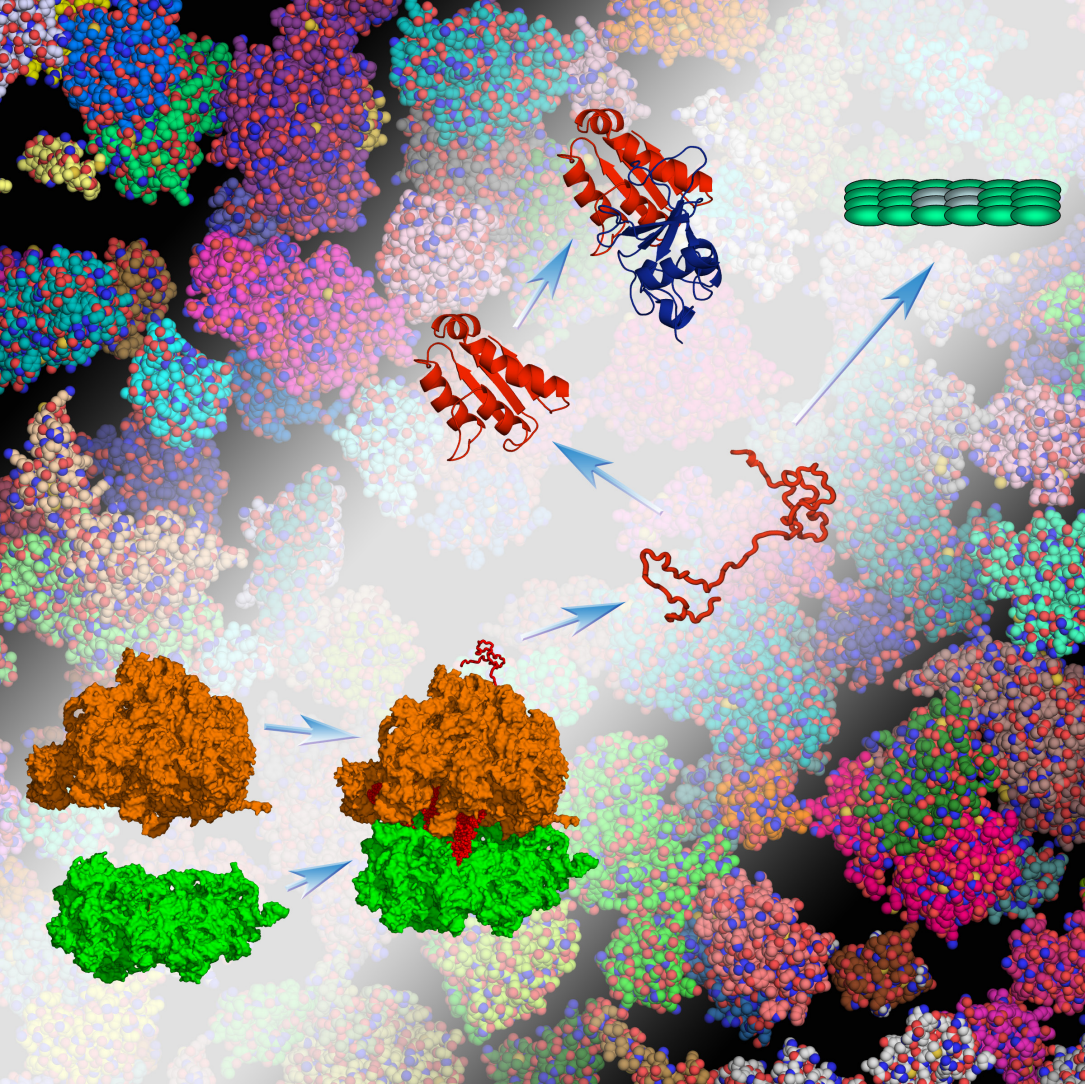
**A composite of processes addressed by biophysics**. The depicted processes include the binding of the large and small subunits of the ribosome, the folding of a nascent protein, its binding to another protein, and its aggregation. The equilibrium constants and rate constants of these processes can be computed according to basic theories of physical chemistry, and can be changed by many orders of magnitudes by the structures, dynamics, and interactions of the constituent molecules. It should also be recognized that these biophysical properties in the crowded native environment can differ significantly from those determined under dilute conditions of typical *in vitro *experiments (Zhou *et al.*, *Annu Rev Biophys *2008, **37**:375-397).

Indeed. Many biophysical concepts, theories, and tools were originally developed in physical chemistry. Binding affinity, key to characterizing specificity and selectivity in molecular interactions, derives from equilibrium constants of chemical reactions. Rate theories and the stopped-flow technique for measuring rate constants are other examples. However, in adapting these physical chemistry concepts and theories to biology, it is important to recognize the much higher complexities of biomolecules and their native environments (Figure 1).

Many biophysicists have focused on biology at the molecular level, but more and more of them are now studying processes at the cellular level. For example, the National Cancer Institute has funded 12 Physical Sciences-Oncology Centers, where physicists and cancer biologists are teamed up to uncover the physical principles that govern the emergence of cancer and its behavior at different scales.

## I want to purse a career in life sciences. Are there reasons that I should study biophysics rather than directly go to biology?

Tackling the challenging biological problems of the future will require ever closer integration of biology and physics in advancing new concepts and new experimental techniques. A life scientist with a solid training in physics will have unique strengths in this integration.

Research at the intersection of the physical and life sciences is full of opportunities. These have been targeted by the National Institutes of Health and the National Science Foundation as well as by the Burroughs Wellcome Fund.

## Although studying similar biological problems, some describe themselves as biochemists while others describe themselves as biophysicists. How come?

The blurring of the disciplinary boundary is a good sign! That said, at present most people doing biological research have been trained in traditional departments. As a result, there are still cultural differences. For example, a biochemist may be interested in reducing a complex biological process such as protein synthesis into a sequence of binding events and chemical reactions, whereas a biophysicist may be interested in the rate constants of these events. So the biochemist identifies the constituent molecules and frames the biologically interesting questions, and the biophysicist then asks how do I explain the biochemical observations based on the structures and the interactions of the constituent molecules? Both are needed to discover how the biological process actually works.

## What are the most important directions in 21^st ^century biophysics?

**Figure 2:**
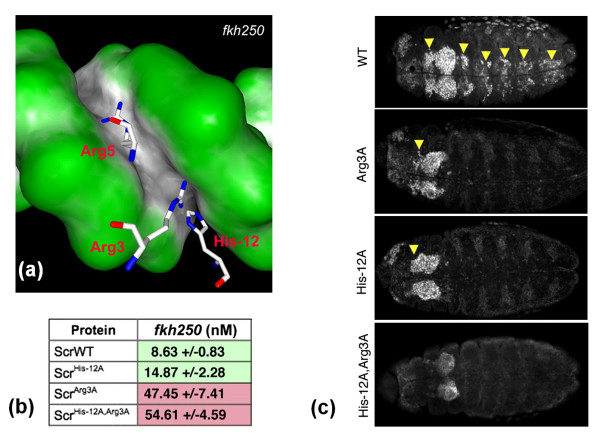
**A small difference in intermolecular binding affinity may change organismal phenotype**. **(a) **The interaction of three basic residues of a *Drosophila *Hox protein, Sex combs reduced (Scr), with the minor groove of fkh250 DNA. **(b) ***K*_D _values of wild-type Scr and three alanine mutants binding fkh250. **(c) ***Drosophila *embryos ubiquitously expressing ScrWT and Scr mutants. Arrowheads indicate formation of salivary glands. Reprinted from *Cell*, volume 131, R Joshi, JM Passner, R Rohs, R Jain, A Sosinsky, MA Crickmore, V Jacob, AK Aggarwal, B Honig, and RS Mann, Functional specificity of a Hox protein mediated by the recognition of minor groove structure, pages 530-543, copyright (2007), with permission from Elsevier.

One clear trend is that biology is becoming more and more quantitative. This trend is well justified, since, for example, even a six-fold decrease in DNA-binding affinity of a mutant protein may be responsible for a change in phenotype (Figure 2). After sequences and structures, the next frontier may be the determination of binding affinities and rate constants. Biophysics will undoubtedly play a prominent role in pushing this frontier. Ultimately it may be possible to compute the binding affinities and rate constants of all elementary biological steps (Figure 1). And it is important to recognize that cellular processes are stochastic in nature.

As knowledge at the molecular level expands, studies at the network and cellular levels will come into focus. Here again biophysics will make unique, important contributions, in terms of new concepts, new theories, and new experimental and computational tools.

In the 21^st ^century, dramatic progress can be anticipated in early diagnoses and treatments of cancer, Alzheimer's, and other diseases, in the development of biologically inspired materials, devices, and energy sources, and in understanding how the human brain works. In each of these endeavors, biophysics will be a central player.

These directions paint a tantalizing future for biophysics. Still, given that modern physics came out of revolutionary (not evolutionary!) developments in the early 20^th ^century, one cannot help but wonder: does another revolution await biophysics?

## Where can I find out more?

### Books

Crick F: *What Mad Pursuit: A Personal View of Scientific Discovery*. New York: Basic Books; 1988.

National Research Council (US) Committee on Forefronts of Science at the Interface of the Physical and Life Sciences: *Research at the Intersection of the Physical and Life Sciences*. Washington, DC: National Academies Press; 2010.

### Articles

Knight J: **Bridging the cultural gap**. *Nature *2002, **419**:244-246.

Phillips R, Quake SR. **The biological frontier of physics**. *Phys Today *2006, **59**:38-43.

### Websites

Physical Sciences-Oncology Centers: http://physics.cancer.gov/

New Biomedical Frontiers at the Interface of the Life and Physical Sciences: http://grants.nih.gov/grants/guide/pa-files/PAR-10-142.html

Career Awards at the Scientific Interface: http://www.bwfund.org/pages/129/Career-Awards-at-the-Scientific-Interface/

